# Short-Term Functional Outcome and Satisfaction Rate of Unicompartmental Knee Arthroplasty in Indian Population: A Retrospective Study

**DOI:** 10.7759/cureus.9033

**Published:** 2020-07-06

**Authors:** Debashish Mishra, Saurav N Nanda, Saswat Samant, Sumanyu K Tripathy, Ashok Gachhayat, Govind V J

**Affiliations:** 1 Orthopaedics, Kalinga Institute of Medical Sciences, Bhubaneswar, IND; 2 Orthopaedics, Apollo Hospital, Bhubaneswar, IND

**Keywords:** medial compartmental osteoarthritis, functional outcome, satisfaction rate, unicondylar arthroplasty

## Abstract

Introduction: Unicompartmental knee arthroplasty (UKA) is a procedure used to treat isolated medial or lateral compartmental osteoarthritis of the knee joint. This procedure involves retention of cruciate ligaments which leads to better functional outcome due to preservation of normal kinematics of the knee joint. In the Indian population, due to requirement of squatting and cross leg sitting habits, knee with more range of movement and with good kinematics is a required feature. The study aims to observe the functional outcome, mortality, revision rate, length of hospital stay and satisfaction rate in two-year postoperative patients in a tertiary health care centre.

Methods: A total of 17 knees of 15 patients were recruited for the study after applying strict inclusion and exclusion criteria. These patients were operated during the period from March 2015 to March 2018. Ten female patients (67%) and five males (33%) were included. The average age was 61 years. All operations were performed by a single surgeon, with a similar implant from a single company. Similar protocol was used both for surgical technique (minimal invasive) and postoperative rehabilitation for all the patients.

Results: The functional outcome in the form of Oxford Knee Score (OKS) and Euro-Quol (EQ-5D) scores improved significantly in all the patients at one year postoperatively, and the improvement remained significant for two years. Satisfaction rate was 91.7% (SD-12.8) at two years. Except for one patient (5.8%), all patients were able to cross leg and squat easily. At the end of two years, the overall survival rate of the implant was found to be 94.1%.

Conclusion: The unicondylar arthroplasty provides excellent satisfaction to the appropriately selected patients with good survivorship of implant. It can be a surgery of choice for Indian population as it restores normal kinematics of knee joint and allows the patient to cross leg and squat with a more range of movement.

## Introduction

Unicompartmental knee arthroplasty (UKA) is a procedure used to treat isolated medial or lateral compartmental osteoarthritis with fully correctable varus deformity and with less than 15 degrees of fixed flexion deformity of the knee joint [1]. Since it was introduced in 1982, it has consistently demonstrated better survivorship of 94%-100% at 10 years, 95% at 14 years and 90% at 15 years in multiple studies [2-7]. It is a well-recognized procedure with an added advantage over total knee arthroplasty in terms of a smaller incision, shorter hospital stay and lesser tissue dissection [8,9]. This procedure involves the retention of cruciate ligaments which leads to better functional outcome due to normal kinematics of the knee joint [10]. The morbidity and mortality rates are also low in comparison to total knee arthroplasty [11-14].

In the Indian population, due to squatting and cross leg sitting habits, knee with more range of movement and with good kinematics is a desirable feature. There are very few studies on the functional outcome of UKA in both Indian population and abroad [15,16]. The aim of the study is to observe the functional outcome, mortality, revision rate, length of the hospital stay and satisfaction rate of two-year postoperative patients, operated in our tertiary health care centre.

## Materials and methods

This study was a retrospective study conducted in our tertiary health care centre. All the persons who were operated for unicondylar knee arthroplasty for isolated medial compartmental osteoarthritis with a minimum follow-up of two years were included in the study. The patients with incomplete medical records and those who denied for proper consent were excluded from the study.

**Figure 1:**
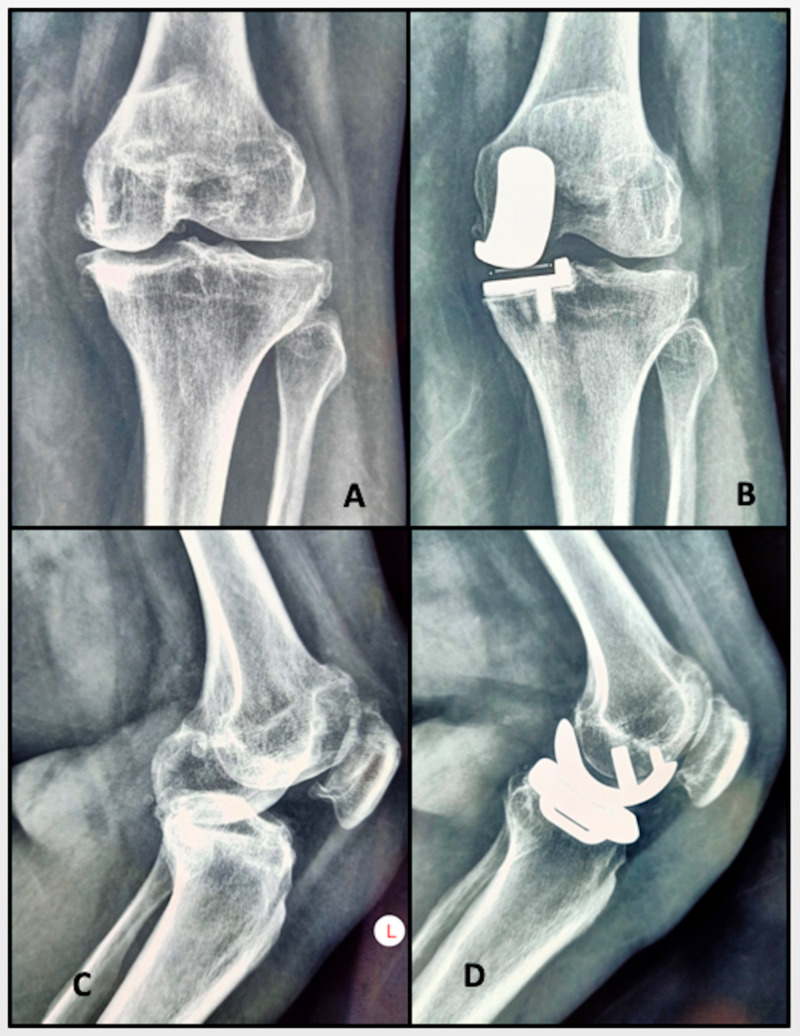
Preoperative and postoperative radiographs (A) Preoperative AP radiograph of the left knee showing medial compartmental osteoarthritis. (B) Postoperative AP radiograph of the left knee showing a well-fixed UKR prosthesis. (C) Preoperative lateral radiograph of the left knee. (D) Postoperative lateral radiograph of the left knee with the implant in situ. AP, anteroposterior; UKR, unicondylar knee replacement

**Figure 2:**
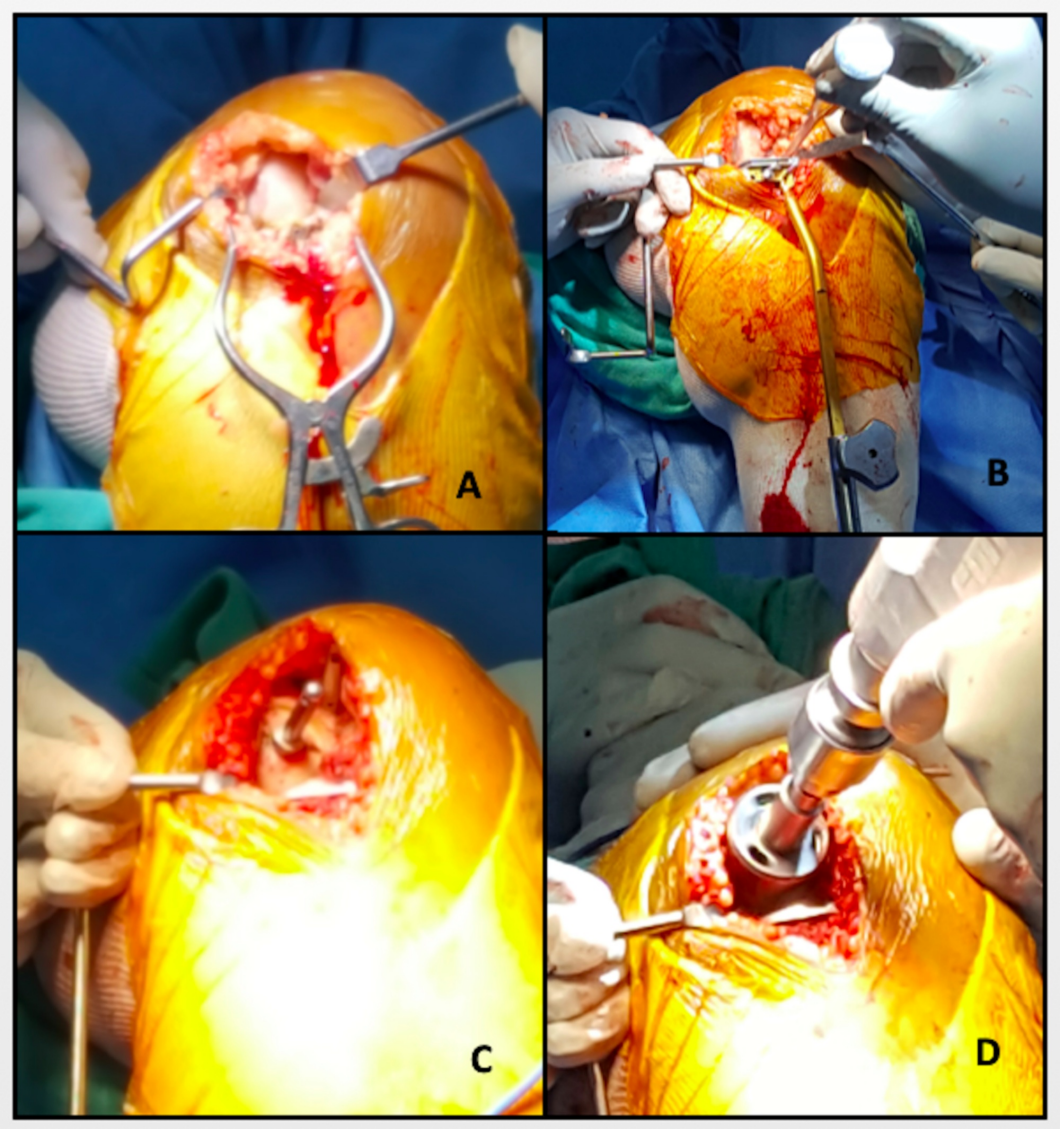
Intraoperative images (A) Skin incision and exposure of the medial compartment of the knee. (B) Placement of tibial jig to tibia cut. (C) Placement of femoral jig. (D) Preparation of femoral side.

A total of 17 knees of 15 patients were considered for the study after applying strict inclusion and exclusion criteria operated from March 2015 to March 2018. There were 10 female patients (67%) and five male patients (33%) in our study. The average age was 61 years. There were 10 knees operated on the right side (59%) and seven knees operated on the left side (41%). All surgeries were performed by a single surgeon, with a similar implant Oxford (Zimmer Biomet, Warsaw, IN, USA) partial Knee replacement system (Figure 1). A similar protocol was used both for surgical technique (standard minimal invasive technique) and standard postoperative rehabilitation for all the patients (Figure 2).

We have used patient-reported outcome measures, including Oxford Knee Score (OKS) and Euro-Quol (EQ-5D) score, to analyze the functional outcome [17]. Similarly, we used a numerical scale score tool to analyze satisfaction rate along with the ability for sitting cross-leg and squatting. All data were evaluated preoperative, one-year postoperative period and two-year postoperative period. Statistical analysis was performed on SPSS software of version 20 (IBM Corporation, Armonk, NY, USA). A p-value of less than 0.05 was considered statistically significant.

## Results

The functional outcome in the form of OKS and EQ-5D scores improved significantly (p-value = 0.02) in all the patients at one year of postoperative time, and the improvement remained significant in two years. The preoperative, one-year postoperative and two-year postoperative OKS were 20.3, 36.7 and 38.9, respectively (Table 1).

**Table 1:** Oxford Knee Score (OKS)

	Preoperative OKS	One-year postoperative OKS	Two-year postoperative OKS
OKS score	20.3	36.7	38.9

Similarly, the EQ-5D scores improved significantly (p-value = 0.01) in all the patients at one year of postoperative time as compared to preoperative scores. The preoperative, one-year postoperative and two-year postoperative EQ-5D scores were 0.4, 0.8 and 0.8, respectively (Table 2).

**Table 2:** Euro-Quol (EQ-5D) Score

	Preoperative EQ-5D	One-year postoperative EQ-5D	Two-year postoperative EQ-5D
EQ-5D score	0.4	0.8	0.8

**Figure 3:**
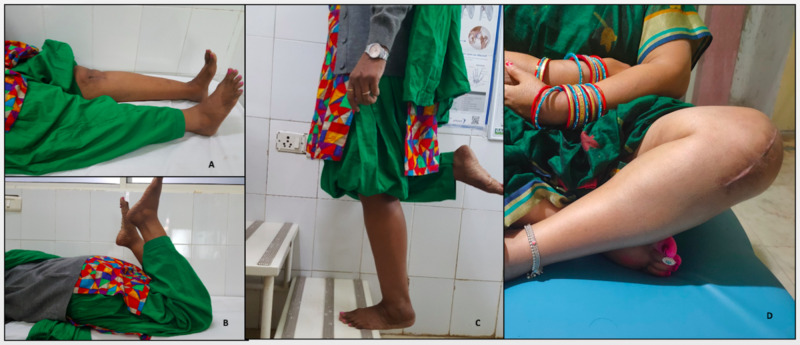
Four-month postoperative clinical results (A) Postoperative achievement of full extension. (B) Postoperative achievement of flexion of 130 degrees. (C) Postoperative achievement of one-leg stand on the operative side. (D) Postoperative achievement of cross leg sitting.

The satisfaction rate was 91.7% (SD-12.8) at two years. The length of hospital stay was 4.9 days (4-9 days). One patient (5.8%) had a history of fall, and there was a dislocation of the knee and was operated for total knee replacement. The overall survival rate of the implant of our series was found to be 94.1% at two years. Except for one patient (5.8%), all patients could able to sit cross leg and squat easily (Figure 3). There was no mortality in our study.

## Discussion

The overall survival rate of our study was 94.1% and was comparable to other studies present in the scientific literature. Pandit et al. in a study of 688 knees in seven years found that the survival rate was 97.3% [18]. Carr et al. showed a 99% survivorship in 121 knees at a follow-up of 3.8 years [19]. Similarly, the designer surgeon Goodfellow and his colleagues reported a survivorship of 91% in 103 knees with five years of follow-up [20]. A 98% survival was reported by Murray et al. in 143 knees with 7.6 years of follow-up [2]. A survivorship at a maximum of 10 years was found to be 91% in the study conducted by Price et al. with 52 patients [21]. Even Keys et al. reported a 100% survival rate of the UKA implant at 10 years after studying his 40 knees [3]. Multiple independent studies showed reasonable survival rates of UKA in both short-term and long-term study. Vorlat et al. documented 93% survival rates of 41 patients at five-year follow-up [22], Emerson et al. noted a 93% survival rate at 10 years in 50 knees [23]. Kort et al. reported a 96% survival rate up to six years in 46 knees [24]. However, most of the study failed to mention the functional outcome of UKA in their study.

We analyzed our functional outcome in the form of Oxford Knee Society scores and EQ-5D scores in this study, and the results were comparable with other studies available in the scientific literature [18,19,25]. Excellent Oxford scores of 40.1 were achieved by Carr et al. in his study in 121 patients with a mean follow-up of 3.8 years [19]. The Oxford score of 38 with a mean follow-up of five years of 29 patients was reported by Langdown et al. [26]. Oxford scores of 38.3 in 78 patients with two-year follow-up were reported by Luscombe et al. in his study [25]. Pandit et al. in his study found Oxford scores of 39 with his technique of minimally invasive approach [18]. Majority of UKA (81.5%) were implanted via minimal invasive procedure and had excellent results when compared with the standard approach, although there was no statistical significance of the functional outcome when compared between two approaches. 

In our study, we have not done any revision surgery because of implant failure. One patient with post-traumatic periprosthetic fracture was operated with total knee arthroplasty in our study population. The revision rate in the designer group of UKA is 2.9% and by the other authors who reported in the scientific literature varies between 4.3% and 6.3% [27,28]. A comprehensive review was done over eight years by Bakers et al. from the data of the National Joint Registry and observed that the five-year implant survival rates were lowest among lowest volume centre [29]. They documented that the surgeons who were operating less than 13 UKA per year have more revision rates than those who were operating more cases per year [29]. 

The limitations of our study were the small sample size and retrospective study. We have included the satisfaction rate along with functional outcome and along with the ability to sit cross leg and squat to address the requirement of the Indian population particularly. We found the unilateral knee replacement is a good option for the patients with unicondylar knee arthritis with an excellent outcome. However, extensive population studies may be required to justify our result further.

## Conclusions

The unicondylar arthroplasty provides excellent satisfaction to the appropriately selected patients with good survivorship of implant. It is a minimally invasive surgery with better functional outcome and lesser complications as compared with total knee arthroplasty. It can be a surgery of choice for Indian population as it restores normal kinematics of knee joint and allows the patient to sit cross leg and squat with a more excellent range of movement.
